# Longitudinal training and assessing consultation competence, a role for self reflection on performance

**DOI:** 10.1007/s40037-012-0028-x

**Published:** 2012-10-19

**Authors:** Harianne H. M. Hegge, Joris J. P. Slaets, Janke Cohen-Schotanus

**Affiliations:** 1 Institute for Medical Education, University Medical Centre Groningen, University of Groningen, PO Box 30001, 9700 RB Groningen, the Netherlands; 2 Department of Internal Medicine/Geriatrics, University Medical Centre Groningen, Groningen, the Netherlands; 3 Center for Research and Innovation in Medical Education, University Medical Centre Groningen, University of Groningen, Groningen, the Netherlands

**Keywords:** Consultation competence, Self reflection, Assessment, Longitudinal

## Abstract

Medical consultation (patient–doctor encounter), consisting of history taking, physical examination and treatment, is the starting point of any contact between doctor and patient. Learning to conduct a consultation is a complex skill. Both communicative and medical contents need to be applied and integrated. Conducting an adequate consultation is a skill which is gradually learned and perfected during training and career. This article discusses the background and implementation of a longitudinal integrated consultation training programme in clerkships. In the programme, the student’s reflection on the consultation plays an important role in education and assessment.

## Introduction

Medical consultation, also called patient–doctor encounter, is the foundation of health care. Conducting an adequate consultation is gradually learned and perfected during medical training and career [1, 2]. It is a complex skill. Medical, communicative-interactive and contextual components with varying degrees of complexity must be appropriately integrated [3, 4]. Integration of subskills and adding complexity can be accomplished by means of learning in cyclical processes consisting of skills practice, rehearsal, performance and assessment [4]. With respect to learning, students have shared and individual points of attention. Especially the individual learning points are not always taken into account in educational programmes [5]. This article focuses on how to teach students to conduct medical consultation. We describe a programme which integrates the above-mentioned principles -skills integration, complexity and individual learning points- and report our findings. We use self-reflection on performance as a source for learning and assessing clinical consultation skills.

## Longitudinal training programme regarding consultation competence

**Fig. 1:**
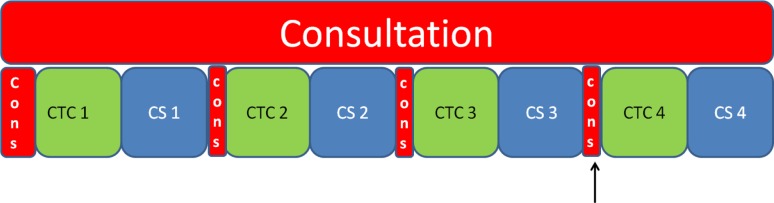
Schematic representation of the educational elements of the first year of the clerkships (M1). *Top* consultation training takes place throughout the year. *Bottom* representation of setting for training = programme in time (chronological). *cons* full weeks of training in consultation competence (2 weeks, 1 week, 1 week, 1 week). *CTC* clinical training centre (5 weeks). *CS* clerkship (hospital, 5 weeks). *Arrow* time of consultation test

During the first year of the Master at the medical faculty of the University of Groningen, the Netherlands, skills training and clerkships alternate during four junior rotations (Fig. 1) [6]. Following the principle of just-in-time learning, those clinical skills are taught in the training period relevant to the subsequent clerkships (e.g. wound treatment in the training period prior to surgery clerkship).

Part of the dual learning programme is a longitudinal programme aimed at training medical consultation: five one-week courses which specifically focus on learning how to conduct a consultation (Fig. 1).

In the four training periods students practise with simulated patients (recorded consultations) and receive feedback from peers and teachers (MDs). The recordings are also used to: [1] draw up patient charts to show their clinical reasoning skills; [2] reflect on strengths and weaknesses; and [3] formulate learning points. There are small-group sessions where individual development areas noticed during the preceding clerkships are reflected on and discussed.

During the training period students can practise their consultation learning points, after which they can continue practising their consultation competence on actual patients in a clerkship. The consultation training programme consists of four cycles of theory and practice in simulated and real settings with interim evaluation and feedback. An assessment is taken in the last cycle.

Medical consultation is examined by means of a log, which records the progress made during the year, and a consultation skill test.

The assessment of consultation skill is threefold: conducting a complete medical consultation with a simulated patient (recorded on DVD), writing a self-reflective report about the consultation, and writing a complete medical chart. The student has allocated time to view his own recording, and to write the chart and the self-reflective report. In the self-reflective report the student adds comments on his performance and his reflection on it (Box 1).

**Box 1:** Examples of fragments from reflective reports

I found the physical examination difficult. I was not sure what to do
In practice I would have asked better differentiating questions. Although I asked a couple of questions, I noticed that I had to dig deep to come up with some more
When reviewing the consultation, I saw that there were times when an emotional reflection would have been better. E.g.…
In general I found that I was empathetic and concerned about the fears of the patient

Assessors (MDs) rate the recording, chart and reflection on a three-point scale (insufficient; satisfactory; excellent) and give an overall grade of the student’s ability to perform an adequate medical consultation. The assessor also provides feedback, positive as well as points of improvement. Blind reassessment by a second assessor takes place when the first is in doubt. The assessors use global assessment criteria (Box 2).

**Box 2:** Assessment criteria

Recording/DVD	
Communication	Direction, communication skills and interaction with patient
Medical content	Choice and execution of physical examination, logical (working) diagnosis and taking appropriate therapeutic management
Execution within time	
Chart	
	In accordance with conducted consultation
Reasoning	Diagnostic and therapeutic, proper argumentation for differential diagnosis and proper argumentation for diagnostic and therapeutic management
	Mentioned data and findings are relevant for diagnostic process
Reflection	
	Insight into own strengths and weaknesses; matching conducted consultation

All assessors are trained and take additional training twice a year.

## Evaluation

On an annual basis, about 60 % of the tests are marked as satisfactory, 25 % as excellent and 15 % as insufficient. In the event of an insufficient grade, the student can, depending on the reason, resit or get a customized solution. In 1–2 % of the students there are major problems mainly concerning communication and/or incorrect performance of the physical examination. Examples of these communication problems are: not being in control, insufficient contact with the patient or being inaccurate. The reflective report clarifies whether the student is aware of these problems.

The faculty student evaluation revealed that more than 70 % of the students were satisfied with ‘sufficient time for practising’, ‘effective methods used’, ‘expert teachers’, ‘recording with a simulated patient was informative and of good quality’, ‘there was sufficient connection of the test to education’ and they felt ‘well prepared to conduct medical consultations in the next clerkships’.

Points of improvement were: the ‘distribution of hours in the week’, the ‘debriefing of the contact with the simulation patient’ (there was a greater need for feedback on medical content), ‘the consultation log’ and the ‘assessment of the test’ (result is not a correct reflection of students’ abilities).

## Discussion

The main objective of our longitudinal training programme is to teach students to perform medical consultation independently and to a satisfactory degree in the remainder of the clerkships. Considering the assessment results and students’ response (they feel well prepared), this objective has been achieved. The structure of the programme allows students to learn consultation skills integrated from the start. The cyclical process with skills practice, rehearsal, performance and assessment as components to learn complex skills [4] is well recognized in the cycles of theory and practice in simulated and real settings with interim evaluation and feedback.

Literature shows that observed assessments are not done often enough in clerkships [7, 8]. The importance of these assessments becomes clear on closer examination of the problem students. It turns out (whether consciously or not) that these students were often less frequently observed in the clinic when doing a consultation than others. The chart was discussed but the performance was not observed. Because the consultations of the training centre are recorded the teachers can do a ‘direct observation’ [9] and assess them at a convenient time.

The student is forced to reflect on his own progress by interim evaluations. The teacher and peers give an evaluation of the student’s performance and his reflection, so the student can see if his self-assessment is realistic and his performance up to professional standards. Literature shows that shared reflection is better than an individual one [10]. It motivates learning by giving different perspectives. The student’s thoughts on the consultation can be compared with those of an expert. At the same time, the supervisor receives insight into the behaviour and thoughts of the student. If this insight does not exist, it is not clear whether the student understands and whether he will be able to transfer his experiences to other domains of health care and personal learning needs [11, 12].

The teacher is in our case the gold standard for assessment. This can create a potential source of bias. We try to avoid this by training the teachers how to use the assessment criteria.

Reflection is an important aspect of our training programme. By consequently repeating reflection in training and assessing, looking back becomes a habit and with that a basis for lifelong learning [10]. Shortcomings can be identified to guide personal learning needs. Lifelong learning is most efficient when personal learning objectives are met [2, 5].

Upon examination, the teachers can directly observe the competence of the student, read the clinical reasoning in the chart and get an impression on whether the student’s evaluation of his own consultation is the same as that of the experienced teacher. Also, if need be, the teacher can watch the video again, together with the student. Furthermore, the student can indicate in the reflective report that he understands the test and knows how he should proceed. The test is well-structured with respect to time and scope of the problem and reasonable to standardize because of the use of simulated patients [13]. The assessment takes into account the total consultation. If the differential diagnosis and the diagnostic policy afterwards correspond with the consultation and the medical problem, this is a satisfactory consultation even if the exact diagnosis was not found.

A point of attention is that some students question whether the rating of the test accurately reflects reality. A possible explanation for this may be that students are accustomed to getting high grades in their clerkships and therefore get a different picture of their abilities. The average grade for a consultation in the clerkships is around eight out of ten (excellent). ‘Overly generous performance evaluations’ are a known phenomenon in work-based assessments [14].

The use of simulated patients helps to standardize the test, but has the disadvantage that the setting is not authentic. For some students, it is difficult to make a good estimate of the complaint without a realistic clinical situation. If this had any influence on the consultation, it was generally well marked in the reflective report and thus taken into account. Studies comparing simulated patients with real patients mostly found no difference between performance of skills [13, 15] and the general instructiveness of simulated patients was as high as that of real patients [16].

## Conclusion

The main objectives of our programme were teaching students to conduct a consultation by integrating skills, complexity and individual learning points in order to lay a foundation for lifelong (autonomous) learning. By adding a personal strength-weaknesses analysis and a reflective report to the consultation test, we encouraged students to reflect on their own performance and view learning as a continuous process of improvement. With this we managed to set up a course in which the above-mentioned goals have been qualitatively achieved. Further research is ongoing to quantitatively substantiate this.

## Essentials

Consultation competence can be learned:

- as an integrated skill
- on a longitudinal basis
- with an important educational and selective role for self-reflection.
